# (*E*)-*N*′-(2,3,4-Trihy­droxy­benzyl­idene)­isonicotinohydrazide dihydrate

**DOI:** 10.1107/S1600536810043965

**Published:** 2010-10-31

**Authors:** H. S. Naveenkumar, Amirin Sadikun, Pazilah Ibrahim, Jia Hao Goh, Hoong-Kun Fun

**Affiliations:** a School of Pharmaceutical Sciences, Universiti Sains Malaysia, 11800 USM, Penang, Malaysia; b X-ray Crystallography Unit, School of Physics, Universiti Sains Malaysia, 11800 USM, Penang, Malaysia

## Abstract

In the title isoniazid derivative, C_13_H_11_N_3_O_4_·2H_2_O, the Schiff base mol­ecule exists in an *E* configuration with respect to the acyclic C=N bond. An intra­molecular O—H⋯N hydrogen bond forms a six-membered ring, producing an *S*(6) ring motif. The essentially planar pyridine ring [maximum deviation = 0.0119 (8) Å] is inclined at a dihedral angle of 7.30 (4)° with respect to the benzene ring. In the crystal, inter­molecular O—H⋯N, O—H⋯O, N—H⋯O and C—H⋯O hydrogen bonds link the mol­ecules into two-dimensional arrays lying parallel to the (10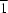) plane. These arrays are further inter­connected into a three-dimensional extended network *via* O—H⋯O and C—H⋯O hydrogen bonds. A weak inter­molecular π–π inter­action [centroid-to-centroid distance = 3.5627 (5) Å] is also observed.

## Related literature

For general background to and applications of the title isoniazid derivative, see: Janin (2007 ▶); Kahwa *et al.* (1986 ▶); Maccari *et al.* (2005 ▶); Slayden & Barry (2000 ▶). For the preparation of the title compound, see: Lourenço *et al.* (2008 ▶). For closely related isoniazid structures, see: Naveenkumar *et al.* (2009 ▶, 2010*a* ▶,*b* ▶,*c* ▶); Shi (2005 ▶). For hydrogen-bond ring motifs, see: Bernstein *et al.* (1995 ▶). For the stability of the temperature controller used for the data collection, see: Cosier & Glazer (1986 ▶).
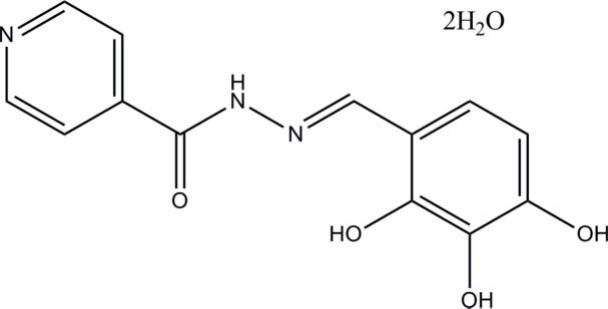


## Experimental

### 

#### Crystal data

C_13_H_11_N_3_O_4_·2H_2_O
*M* *_r_* = 309.28
Monoclinic, 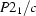

*a* = 6.9504 (5) Å
*b* = 19.9077 (13) Å
*c* = 10.0930 (7) Å
β = 106.416 (2)°
*V* = 1339.60 (16) Å^3^
*Z* = 4
Mo *K*α radiation
μ = 0.12 mm^−1^
*T* = 100 K
0.35 × 0.18 × 0.09 mm

#### Data collection

Bruker APEXII DUO CCD area-detector diffractometer
Absorption correction: multi-scan (*SADABS*; Bruker, 2009 ▶) *T*_min_ = 0.959, *T*_max_ = 0.989
21520 measured reflections
5656 independent reflections
4668 reflections with *I* > 2σ(*I*)
*R*_int_ = 0.031

#### Refinement

*R*[*F* ^2^ > 2σ(*F* ^2^)] = 0.038
*wR*(*F* ^2^) = 0.113
*S* = 1.03
5656 reflections
259 parameters
All H-atom parameters refined
Δρ_max_ = 0.50 e Å^−3^
Δρ_min_ = −0.26 e Å^−3^

### 

Data collection: *APEX2* (Bruker, 2009 ▶); cell refinement: *SAINT* (Bruker, 2009 ▶); data reduction: *SAINT*; program(s) used to solve structure: *SHELXTL* (Sheldrick, 2008 ▶); program(s) used to refine structure: *SHELXTL*; molecular graphics: *SHELXTL*; software used to prepare material for publication: *SHELXTL* and *PLATON* (Spek, 2009 ▶).

## Supplementary Material

Crystal structure: contains datablocks global, I. DOI: 10.1107/S1600536810043965/rz2508sup1.cif

Structure factors: contains datablocks I. DOI: 10.1107/S1600536810043965/rz2508Isup2.hkl

Additional supplementary materials:  crystallographic information; 3D view; checkCIF report

## Figures and Tables

**Table 1:** Hydrogen-bond geometry (Å, °)

*D*—H⋯*A*	*D*—H	H⋯*A*	*D*⋯*A*	*D*—H⋯*A*
O2—H1O2⋯N3	0.883 (18)	1.854 (18)	2.6561 (9)	150.1 (16)
O3—H1O3⋯N1^i^	0.869 (19)	1.835 (19)	2.6911 (10)	168.4 (18)
O4—H1O4⋯O1*W*	0.895 (18)	1.769 (18)	2.6559 (9)	170.5 (18)
N2—H1N2⋯O2*W*^ii^	0.890 (16)	2.132 (15)	2.9910 (9)	162.1 (14)
O1*W*—H1*W*1⋯O1^iii^	0.926 (18)	1.880 (18)	2.7834 (9)	164.6 (16)
O1*W*—H2*W*1⋯O2*W*	0.837 (19)	2.077 (18)	2.8980 (11)	167.0 (18)
O2*W*—H1*W*2⋯O2^iv^	0.831 (15)	2.161 (15)	2.9570 (11)	160.3 (14)
O2*W*—H2*W*2⋯O4^v^	0.86 (2)	1.91 (2)	2.7688 (9)	173.3 (19)
C4—H4*A*⋯O1^vi^	0.922 (14)	2.575 (14)	3.2930 (11)	135.1 (11)
C7—H7*A*⋯O2*W*^ii^	0.988 (14)	2.347 (14)	3.2176 (10)	146.7 (12)

Symmetry codes: (i) 
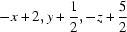
; (ii) 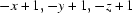; (iii) 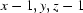; (iv) 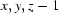; (v) 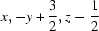; (vi) 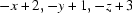.

**Fig. 1.**
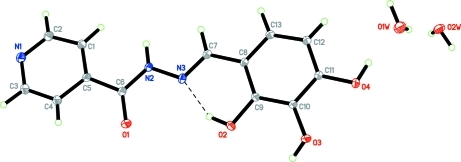
The asymmetric unit of the title isoniazid derivative, showing 50% probability displacement ellipsoids for non-H atoms and the atom-numbering scheme. An intramolecular hydrogen bond is shown as dashed line.

**Fig. 2.**
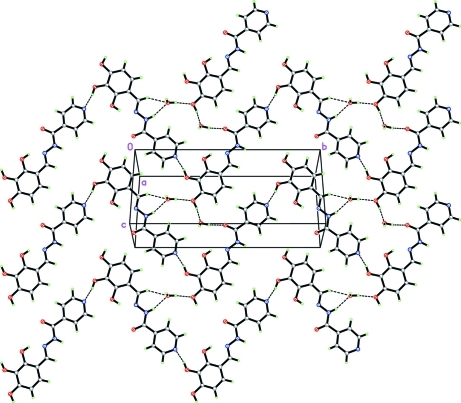
Part of the crystal structure, viewed along the [101] direction, showing a hydrogen-bonded 2D array. Intermolecular hydrogen bonds are shown as dashed lines.

**Fig. 3.**
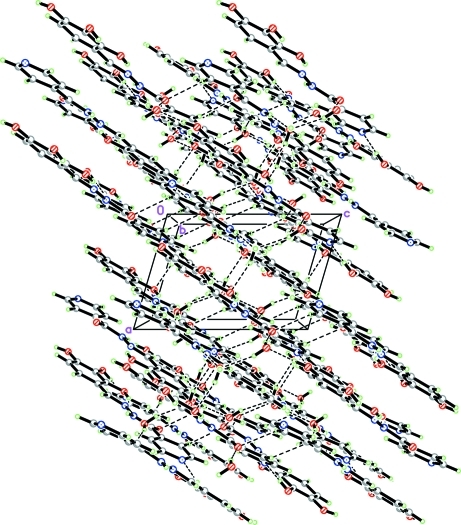
The crystal structure of the title derivative, viewed along the b axis, showing the 2D arrays being linked into a 3D extended network. Intermolecular hydrogen bonds are shown as dashed lines.

## supplementary crystallographic information

### Comment

In the search of new compounds, isoniazid derivatives have been found to possess potential tuberculostatic activity (Janin, 2007; Maccari *et al.*, 2005; Slayden & Barry, 2000). Schiff bases have attracted much attention because of their biological activity (Kahwa *et al.*, 1986). As a part of our current work on synthesis of (*E*)-*N*'-substituted isonicotinohydrazide derivatives, in this paper we present the crystal structure of the title compound.

The title isoniazid derivative comprises of a (*E*)-*N*'-(2,3,4-trihydroxybenzylidene)isonicotinohydrazide molecule and two water molecules of crystallization (Fig. 1). The Schiff base molecule exists in an *E* configuration with respect to the acyclic C7═N3 bond [C7═N3 = 1.2921 (10) Å; torsion angle N2—N3—C7—C8 = 178.85 (7)°]. An intramolecular O2—H1O2···N3 hydrogen bond (Table 1) generates a six-membered ring, producing an *S*(6) ring motifs (Bernstein *et al.*, 1995). The pyridine ring with atom sequence C1/C2/N1/C3/C4/C5 is essentially planar, with a maximum deviation of 0.0119 (8) Å at atom C5. There is a slight inclination between the pyridine and benzene rings, as indicated by the dihedral angle formed of 7.30 (4)°. All bond lengths and angles are consistent to those observed in closely related isoniazid structures (Naveenkumar *et al.*, 2009, 2010*a*,*b*,*c*; Shi, 2005).

In the crystal packing, water molecules play an extensive part in forming the hydrogen-bonded structure. Neighbouring molecules are linked into two-dimensional arrays parallel to the (101) plane (Fig. 2) by intermolecular O3—H1O3···N1, O4—H1O4···O1W, N2—H1N2···O2W, O1W—H1W1···O1, O2W—H2W2···O4 and C7—H7A···O2W hydrogen bonds (Table 1). These arrays are further interconnected by intermolecular O1W—H2W1···O2W, O2W—H1W2···O2 and C4—H4A···O1 hydrogen bonds (Table 1) into a three-dimensional extended structure (Fig. 3). Weak intermolecular π–π aromatic stacking interactions involving the pyridine and benzene rings [*Cg*1···*Cg*2 = 3.5627 (5) Å, symmetry code: -x + 2, -y + 1, -z + 2] stabilizing the crystal structure.

### Experimental

The isoniazid derivative was prepared following the procedure by Lourenço *et al.*, 2008. The title compound was prepared by the reaction between 2,3,4-trihydroxybenzaldehyde (1.0 eq) with isoniazid (1.0 eq) in ethanol/water. After stirring for 1–3 h at room temperature, the resulting mixture was concentrated under reduced pressure. The residue, purified by washing with cold ethanol and ethyl ether, afforded the pure derivative. The brown-coloured single crystals suitable for X-ray analysis were obtained by recrystallization with ethanol.

### Refinement

All H atoms were located from difference Fourier map and allowed to refine freely with N—H = 0.890 (16), O—H = 0.834 (16)–0.926 (18) and C—H = 0.921 (14)–0.988 (13) Å.

### Crystal data

**Table:**

C_13_H_11_N_3_O_4_·2H_2_O	*F*(000) = 648
*M**_r_* = 309.28	*D*_x_ = 1.534 Mg m^−^^3^
Monoclinic, *P*2_1_/*c*	Mo *K*α radiation, λ = 0.71073 Å
Hall symbol: -P 2ybc	Cell parameters from 7532 reflections
*a* = 6.9504 (5) Å	θ = 2.9–34.5°
*b* = 19.9077 (13) Å	µ = 0.12 mm^−^^1^
*c* = 10.0930 (7) Å	*T* = 100 K
β = 106.416 (2)°	Plate, brown
*V* = 1339.60 (16) Å^3^	0.35 × 0.18 × 0.09 mm
*Z* = 4	

### Data collection

**Table:**

Bruker APEXII DUO CCD area-detector diffractometer	5656 independent reflections
Radiation source: fine-focus sealed tube	4668 reflections with *I* > 2σ(*I*)
graphite	*R*_int_ = 0.031
φ and ω scans	θ_max_ = 34.5°, θ_min_ = 2.3°
Absorption correction: multi-scan (*SADABS*; Bruker, 2009)	*h* = −11→11
*T*_min_ = 0.959, *T*_max_ = 0.989	*k* = −31→31
21520 measured reflections	*l* = −16→14

### Refinement

**Table:**

Refinement on *F*^2^	Primary atom site location: structure-invariant direct methods
Least-squares matrix: full	Secondary atom site location: difference Fourier map
*R*[*F*^2^ > 2σ(*F*^2^)] = 0.038	Hydrogen site location: inferred from neighbouring sites
*wR*(*F*^2^) = 0.113	All H-atom parameters refined
*S* = 1.03	*w* = 1/[σ^2^(*F*_o_^2^) + (0.0651*P*)^2^ + 0.2238*P*] where *P* = (*F*_o_^2^ + 2*F*_c_^2^)/3
5656 reflections	(Δ/σ)_max_ = 0.001
259 parameters	Δρ_max_ = 0.50 e Å^−^^3^
0 restraints	Δρ_min_ = −0.26 e Å^−^^3^

### Special details

**Table:**

Experimental. The crystal was placed in the cold stream of an Oxford Cryosystems Cobra open-flow nitrogen cryostat (Cosier & Glazer, 1986) operating at 100.0 (1)K.
Geometry. All esds (except the esd in the dihedral angle between two l.s. planes) are estimated using the full covariance matrix. The cell esds are taken into account individually in the estimation of esds in distances, angles and torsion angles; correlations between esds in cell parameters are only used when they are defined by crystal symmetry. An approximate (isotropic) treatment of cell esds is used for estimating esds involving l.s. planes.
Refinement. Refinement of F^2^ against ALL reflections. The weighted R-factor wR and goodness of fit S are based on F^2^, conventional R-factors R are based on F, with F set to zero for negative F^2^. The threshold expression of F^2^ > 2sigma(F^2^) is used only for calculating R-factors(gt) etc. and is not relevant to the choice of reflections for refinement. R-factors based on F^2^ are statistically about twice as large as those based on F, and R- factors based on ALL data will be even larger.

### Fractional atomic coordinates and isotropic or equivalent isotropic displacement parameters (Å^2^)

**Table:**

	*x*	*y*	*z*	*U*_iso_*/*U*_eq_	
O1	1.01608 (10)	0.51301 (3)	1.29712 (6)	0.01639 (12)	
O2	0.67646 (10)	0.62875 (3)	0.99244 (6)	0.01723 (13)	
O3	0.48628 (10)	0.72385 (3)	0.80029 (6)	0.01782 (13)	
O4	0.29346 (10)	0.68845 (3)	0.54245 (6)	0.01504 (12)	
N1	1.23127 (11)	0.28684 (4)	1.50037 (7)	0.01687 (14)	
N2	0.86827 (10)	0.44582 (3)	1.11516 (6)	0.01251 (12)	
N3	0.78423 (10)	0.50085 (3)	1.03671 (7)	0.01281 (12)	
C1	1.05976 (13)	0.33042 (4)	1.27584 (8)	0.01494 (14)	
C2	1.14706 (13)	0.27828 (4)	1.36485 (8)	0.01646 (15)	
C3	1.22531 (14)	0.34874 (4)	1.55190 (8)	0.01760 (15)	
C4	1.14150 (13)	0.40355 (4)	1.47177 (8)	0.01499 (14)	
C5	1.05899 (11)	0.39475 (4)	1.32999 (7)	0.01165 (13)	
C6	0.97938 (12)	0.45639 (4)	1.24693 (7)	0.01180 (13)	
C7	0.68132 (12)	0.48824 (4)	0.91109 (8)	0.01247 (13)	
C8	0.58229 (11)	0.54102 (3)	0.81860 (7)	0.01120 (13)	
C9	0.58328 (11)	0.60855 (4)	0.85957 (7)	0.01149 (13)	
C10	0.48749 (12)	0.65802 (4)	0.76616 (7)	0.01176 (13)	
C11	0.38589 (11)	0.63908 (4)	0.63033 (7)	0.01128 (13)	
C12	0.38052 (12)	0.57192 (4)	0.58943 (7)	0.01307 (13)	
C13	0.47908 (12)	0.52365 (4)	0.68254 (7)	0.01302 (13)	
O1W	0.13931 (13)	0.64535 (3)	0.28519 (7)	0.02289 (15)	
O2W	0.30816 (11)	0.67273 (3)	0.05904 (7)	0.01959 (13)	
H1O2	0.732 (3)	0.5922 (9)	1.0365 (17)	0.041 (4)*	
H1O3	0.582 (3)	0.7387 (9)	0.8694 (18)	0.045 (5)*	
H1O4	0.247 (3)	0.6695 (9)	0.4591 (19)	0.045 (5)*	
H1N2	0.836 (2)	0.4055 (8)	1.0773 (15)	0.034 (4)*	
H1A	1.001 (2)	0.3205 (7)	1.1767 (14)	0.023 (3)*	
H2A	1.146 (2)	0.2338 (7)	1.3281 (14)	0.023 (3)*	
H3A	1.282 (2)	0.3542 (6)	1.6527 (14)	0.021 (3)*	
H4A	1.135 (2)	0.4445 (7)	1.5129 (14)	0.028 (3)*	
H7A	0.665 (2)	0.4412 (7)	0.8788 (15)	0.029 (3)*	
H12A	0.306 (2)	0.5605 (7)	0.4985 (13)	0.020 (3)*	
H13A	0.479 (2)	0.4751 (7)	0.6573 (14)	0.021 (3)*	
H1W1	0.102 (3)	0.6006 (9)	0.2736 (17)	0.043 (4)*	
H2W1	0.206 (3)	0.6521 (8)	0.2292 (17)	0.038 (4)*	
H1W2	0.425 (2)	0.6675 (7)	0.0549 (15)	0.030 (4)*	
H2W2	0.309 (3)	0.7161 (10)	0.0608 (19)	0.049 (5)*	

### Atomic displacement parameters (Å^2^)

**Table:**

	*U*^11^	*U*^22^	*U*^33^	*U*^12^	*U*^13^	*U*^23^
O1	0.0222 (3)	0.0097 (2)	0.0151 (2)	−0.0004 (2)	0.0018 (2)	−0.00091 (18)
O2	0.0237 (3)	0.0122 (2)	0.0108 (2)	0.0021 (2)	−0.0033 (2)	−0.00140 (18)
O3	0.0247 (3)	0.0080 (2)	0.0154 (2)	0.0006 (2)	−0.0029 (2)	−0.00218 (18)
O4	0.0206 (3)	0.0099 (2)	0.0112 (2)	0.00154 (19)	−0.0010 (2)	0.00164 (17)
N1	0.0185 (3)	0.0132 (3)	0.0162 (3)	0.0008 (2)	0.0005 (2)	0.0031 (2)
N2	0.0154 (3)	0.0084 (2)	0.0115 (2)	0.0012 (2)	0.0002 (2)	0.00147 (19)
N3	0.0139 (3)	0.0104 (2)	0.0126 (3)	0.0018 (2)	0.0014 (2)	0.00298 (19)
C1	0.0188 (4)	0.0110 (3)	0.0133 (3)	0.0015 (2)	0.0017 (3)	0.0002 (2)
C2	0.0195 (4)	0.0109 (3)	0.0169 (3)	0.0021 (3)	0.0018 (3)	0.0012 (2)
C3	0.0218 (4)	0.0148 (3)	0.0129 (3)	0.0008 (3)	−0.0006 (3)	0.0023 (2)
C4	0.0179 (4)	0.0121 (3)	0.0125 (3)	0.0008 (2)	0.0003 (2)	0.0006 (2)
C5	0.0121 (3)	0.0097 (3)	0.0121 (3)	0.0005 (2)	0.0017 (2)	0.0012 (2)
C6	0.0127 (3)	0.0103 (3)	0.0115 (3)	0.0006 (2)	0.0021 (2)	0.0009 (2)
C7	0.0146 (3)	0.0095 (3)	0.0126 (3)	0.0010 (2)	0.0026 (2)	0.0011 (2)
C8	0.0128 (3)	0.0088 (3)	0.0110 (3)	0.0007 (2)	0.0018 (2)	0.0008 (2)
C9	0.0129 (3)	0.0097 (3)	0.0101 (3)	0.0001 (2)	0.0004 (2)	−0.0001 (2)
C10	0.0137 (3)	0.0084 (3)	0.0116 (3)	−0.0001 (2)	0.0010 (2)	0.0001 (2)
C11	0.0129 (3)	0.0093 (3)	0.0106 (3)	0.0002 (2)	0.0015 (2)	0.0010 (2)
C12	0.0163 (3)	0.0102 (3)	0.0110 (3)	0.0005 (2)	0.0011 (2)	−0.0003 (2)
C13	0.0165 (3)	0.0093 (3)	0.0117 (3)	0.0006 (2)	0.0015 (2)	−0.0004 (2)
O1W	0.0385 (4)	0.0160 (3)	0.0140 (3)	−0.0034 (3)	0.0071 (3)	−0.0002 (2)
O2W	0.0281 (4)	0.0104 (2)	0.0201 (3)	−0.0019 (2)	0.0065 (2)	−0.0011 (2)

### Geometric parameters (Å, °)

**Table:**

O1—C6	1.2326 (9)	C3—H3A	0.988 (13)
O2—C9	1.3743 (9)	C4—C5	1.3941 (10)
O2—H1O2	0.883 (18)	C4—H4A	0.921 (14)
O3—C10	1.3557 (9)	C5—C6	1.5009 (10)
O3—H1O3	0.868 (18)	C7—C8	1.4442 (10)
O4—C11	1.3577 (9)	C7—H7A	0.987 (15)
O4—H1O4	0.895 (18)	C8—C13	1.4011 (10)
N1—C2	1.3380 (10)	C8—C9	1.4060 (10)
N1—C3	1.3428 (11)	C9—C10	1.3954 (10)
N2—C6	1.3524 (9)	C10—C11	1.4047 (10)
N2—N3	1.3809 (9)	C11—C12	1.3965 (10)
N2—H1N2	0.890 (16)	C12—C13	1.3827 (10)
N3—C7	1.2921 (10)	C12—H12A	0.946 (13)
C1—C5	1.3929 (10)	C13—H13A	1.000 (13)
C1—C2	1.3945 (11)	O1W—H1W1	0.926 (18)
C1—H1A	0.988 (13)	O1W—H2W1	0.838 (18)
C2—H2A	0.960 (14)	O2W—H1W2	0.834 (16)
C3—C4	1.3847 (11)	O2W—H2W2	0.863 (19)
			
C9—O2—H1O2	105.5 (11)	N2—C6—C5	116.10 (6)
C10—O3—H1O3	118.3 (12)	N3—C7—C8	121.57 (7)
C11—O4—H1O4	106.7 (12)	N3—C7—H7A	119.2 (8)
C2—N1—C3	117.41 (7)	C8—C7—H7A	119.2 (8)
C6—N2—N3	118.17 (6)	C13—C8—C9	118.86 (6)
C6—N2—H1N2	124.5 (10)	C13—C8—C7	118.20 (6)
N3—N2—H1N2	117.1 (10)	C9—C8—C7	122.94 (6)
C7—N3—N2	115.84 (6)	O2—C9—C10	117.13 (6)
C5—C1—C2	118.73 (7)	O2—C9—C8	121.91 (6)
C5—C1—H1A	122.3 (8)	C10—C9—C8	120.96 (6)
C2—C1—H1A	118.9 (8)	O3—C10—C9	123.14 (6)
N1—C2—C1	123.29 (7)	O3—C10—C11	118.03 (6)
N1—C2—H2A	117.8 (8)	C9—C10—C11	118.83 (6)
C1—C2—H2A	118.9 (8)	O4—C11—C12	122.13 (6)
N1—C3—C4	123.43 (7)	O4—C11—C10	117.24 (6)
N1—C3—H3A	117.0 (8)	C12—C11—C10	120.63 (6)
C4—C3—H3A	119.6 (8)	C13—C12—C11	119.85 (7)
C3—C4—C5	118.92 (7)	C13—C12—H12A	121.6 (8)
C3—C4—H4A	119.9 (9)	C11—C12—H12A	118.6 (8)
C5—C4—H4A	121.1 (9)	C12—C13—C8	120.85 (7)
C1—C5—C4	118.18 (7)	C12—C13—H13A	122.3 (8)
C1—C5—C6	125.05 (7)	C8—C13—H13A	116.8 (8)
C4—C5—C6	116.76 (6)	H1W1—O1W—H2W1	105.0 (15)
O1—C6—N2	122.73 (7)	H1W2—O2W—H2W2	97.2 (15)
O1—C6—C5	121.16 (7)		
			
C6—N2—N3—C7	179.26 (7)	C13—C8—C9—O2	178.05 (7)
C3—N1—C2—C1	1.77 (14)	C7—C8—C9—O2	−1.23 (12)
C5—C1—C2—N1	−0.22 (14)	C13—C8—C9—C10	−1.49 (12)
C2—N1—C3—C4	−1.57 (14)	C7—C8—C9—C10	179.23 (7)
N1—C3—C4—C5	−0.19 (14)	O2—C9—C10—O3	1.24 (12)
C2—C1—C5—C4	−1.57 (12)	C8—C9—C10—O3	−179.21 (8)
C2—C1—C5—C6	177.13 (8)	O2—C9—C10—C11	−178.29 (7)
C3—C4—C5—C1	1.76 (12)	C8—C9—C10—C11	1.26 (12)
C3—C4—C5—C6	−177.05 (8)	O3—C10—C11—O4	0.08 (11)
N3—N2—C6—O1	−3.53 (12)	C9—C10—C11—O4	179.64 (7)
N3—N2—C6—C5	177.10 (7)	O3—C10—C11—C12	−179.47 (8)
C1—C5—C6—O1	−166.63 (8)	C9—C10—C11—C12	0.09 (12)
C4—C5—C6—O1	12.09 (12)	O4—C11—C12—C13	179.27 (7)
C1—C5—C6—N2	12.74 (12)	C10—C11—C12—C13	−1.20 (12)
C4—C5—C6—N2	−168.54 (7)	C11—C12—C13—C8	0.97 (12)
N2—N3—C7—C8	178.85 (7)	C9—C8—C13—C12	0.35 (12)
N3—C7—C8—C13	177.91 (8)	C7—C8—C13—C12	179.67 (7)
N3—C7—C8—C9	−2.81 (13)		

### Hydrogen-bond geometry (Å, °)

**Table:**

*D*—H···*A*	*D*—H	H···*A*	*D*···*A*	*D*—H···*A*
O2—H1O2···N3	0.883 (18)	1.854 (18)	2.6561 (9)	150.1 (16)
O3—H1O3···N1^i^	0.869 (19)	1.835 (19)	2.6911 (10)	168.4 (18)
O4—H1O4···O1W	0.895 (18)	1.769 (18)	2.6559 (9)	170.5 (18)
N2—H1N2···O2W^ii^	0.890 (16)	2.132 (15)	2.9910 (9)	162.1 (14)
O1W—H1W1···O1^iii^	0.926 (18)	1.880 (18)	2.7834 (9)	164.6 (16)
O1W—H2W1···O2W	0.837 (19)	2.077 (18)	2.8980 (11)	167.0 (18)
O2W—H1W2···O2^iv^	0.831 (15)	2.161 (15)	2.9570 (11)	160.3 (14)
O2W—H2W2···O4^v^	0.86 (2)	1.91 (2)	2.7688 (9)	173.3 (19)
C4—H4A···O1^vi^	0.922 (14)	2.575 (14)	3.2930 (11)	135.1 (11)
C7—H7A···O2W^ii^	0.988 (14)	2.347 (14)	3.2176 (10)	146.7 (12)

Symmetry codes: (i) −*x*+2, *y*+1/2, −*z*+5/2; (ii) −*x*+1, −*y*+1, −*z*+1; (iii) *x*−1, *y*, *z*−1; (iv) *x*, *y*, *z*−1; (v) *x*, −*y*+3/2, *z*−1/2; (vi) −*x*+2, −*y*+1, −*z*+3.
